# Health professional beliefs, knowledge, and concerns surrounding medicinal cannabis – A systematic review

**DOI:** 10.1371/journal.pone.0216556

**Published:** 2019-05-06

**Authors:** Kyle M. Gardiner, Judith A. Singleton, Janie Sheridan, Gregory J. Kyle, Lisa M. Nissen

**Affiliations:** 1 Discipline of Pharmacy, School of Clinical Sciences, Queensland University of Technology, Brisbane, Australia; 2 School of Pharmacy, Faculty of Medical and Health Sciences, University of Auckland, Auckland, New Zealand; Robert Gordon University, UNITED KINGDOM

## Abstract

**Background:**

The number of jurisdictions allowing access to medicinal cannabis has been steadily increasing since the state of California introduced legislation in 1996. Although there is a high degree of legislative heterogeneity across jurisdictions, the involvement of a health professional is common among all. This places health professionals at the forefront of therapy, yet no systematic review of literature has offered insight into the beliefs, knowledge, and concerns of health professionals regarding medicinal cannabis.

**Methods:**

Using a predetermined study protocol, PubMed, EMBASE, PsycINFO, CINAHL, and Scopus databases were searched for studies indexed up to the 1^st^ August 2018. Pre-defined inclusion and exclusion criteria were applied uniformly. Screening for relevancy, full-text review, data extraction, and risk of bias were completed by two independent investigators. Risk of bias was assessed using CASP criteria (qualitative) and a modified domain-based risk assessment tool (quantitative).

**Results:**

Of the 15,775 studies retrieved, 106 underwent full-text review and of these, 26 were included. The overall risk of bias was considered low across all included studies. The general impression was that health professionals supported the use of medicinal cannabis in practice; however, there was a unanimous lack of self-perceived knowledge surrounding all aspects of medicinal cannabis. Health professionals also voiced concern regarding direct patient harms and indirect societal harms.

**Conclusion:**

This systematic review has offered a lens through which to view the existing literature surrounding the beliefs, knowledge, and concerns of health professionals regarding medicinal cannabis. These results are limited, however, by the implicit common-sense models of behaviour utilised by the included studies. Before strategies can be developed and implemented to change health professional behaviour, a more thorough understanding of the factors that underpin the delivery of medicinal cannabis is necessary.

## Introduction

Current literature suggests that cannabis was first used medicinally by the ancient Chinese before spreading throughout the rest of the world.[1, 2] Although there is difficulty discerning medicinal and non-secular use during early periods, it was by anecdote that cannabis was passed down and considered medically useful.[2, 3] Cannabis continued to be used medicinally until the early-20^th^ century.[4] It was this period of time that saw the prohibition of opium and coca leaves with prohibition extending to cannabis due to its psychoactive properties. The penultimate prohibitive effort was the signing of the 1961 United Nations Single Convention on Narcotic Drugs.[5] Under this convention, cannabis was considered to have an extremely limited therapeutic value while simultaneously being considered to be a high risk of dependence and misuse.[5] Subsequently, generations of health professionals were educated in a system where cannabis was considered purely illicit. More recent studies have demonstrated, however, a plausible rationale for therapeutic action.[6–8] Yet, there are difficulties translating these findings into practice due to a number of issues surrounding the creation of evidence. For instance, it has been very challenging to perform clinical trials on herbal cannabis due to the enduring illicit status of cannabis across much of the world. Furthermore, studies performed on pharmaceutical cannabis preparations often lack consistency in the products and composition of cannabinoids used which makes comparison of trial results hard. Among others, these difficulties are commonly acknowledged in studies examining cannabinoid preparations alongside calls for further large, robust randomised controlled trials to confirm the effects of cannabinoids relative to safety and efficacy.[9–12]

A public vote saw the State of California approve the use of medicinal cannabis in 1996.[13] Since this time, many other jurisdictions have legislated for medicinal cannabis.[14] Although all jurisdictions are using different regulatory frameworks, common among all is the involvement of a health professional in the delivery of medicinal cannabis.[15, 16] For purposes of this study, ‘delivery’ includes authorisation and supply. Depending on the jurisdiction, authorisation can include medical recommendations or prescribing, while supply involves the dispensing and administration of medicinal cannabis.[15, 16] It is for this reason that a review of health professionals’ beliefs and knowledge of medicinal cannabis is important. Without the support of these key stakeholders it is likely that many who may benefit from medicinal cannabis will not receive therapy. By working to better understand the broader context in which health professionals deliver medicinal cannabis, further strategies can be devised to change health professional behaviour moving forward.

The principal aim of this study is to systematically review the existing literature surrounding health professional beliefs, knowledge, and concerns within the context of delivering medicinal cannabis. It is important to note that this study is neither positioned in support or against the availability of medicinal cannabis. As per the aim, the position of this study is to strictly report the beliefs, knowledge, and concerns of health professional delivering medicinal cannabis.

## Methods

Using a pre-determined study protocol, a literature search was performed to identify studies considering the beliefs, knowledge, and concerns of health professionals surrounding the delivery of medicinal cannabis. The specific research questions addressed were:

1. How do health professionals feel about the use of medicinal cannabis in clinical practice?
2. How knowledgeable are health professionals regarding medicinal cannabis?
3. What concerns exist for health professionals regarding the delivery of medicinal cannabis

A preliminary review of the literature revealed an inconsistency in the way studies exploring the views and beliefs of health professionals regarding medicinal cannabis were indexed. As such, intentionally simple, yet broad search phrases were developed to ensure all relevant studies were captured (S1 Table). Using the pre-determined search phrases, electronic searches were conducted by the chief investigator (KG) using the PubMed, PsycINFO, CINAHL, Scopus, and EMBASE databases for studies indexed between database inception and the 1^st^ August 2018. Cited reference lists were also examined. Electronic searches were limited to studies involving humans and the English language, before duplicate entries were removed. Screening for relevancy, full-text review, data extraction, and risk of bias evaluation were completed by two independent investigators (KG and JSi). Disagreements were discussed, and consensus reached. If consensus could not be reached, a third investigator (GK) mediated. Full-text review was considered against pre-specified inclusion and exclusion criteria. Inclusion criteria were: (a) health professional participants; (b) the study was concerned with ‘medicinal’ cannabis; (c) the primary behaviour being studied was the delivery of medicinal cannabis. Exclusion criteria were: (a) the study was indexed as an abstract, editorial, commentary, or review; (b) the study was concerned with the ‘recreational’ use of cannabis; (c) non-professional or mixed professional-patient samples. Data were extracted from studies into a pre-designed review form under the following categories: first author and publication year, study design, country/state, target population, sample size, sampling methods, data collection methods, response rate, whether the data collection tool was piloted, outcome measures, and main findings.

The method of evaluating bias varied according to each individual study’s design. The Critical Appraisal Skills Programme (CASP) criteria tool was used to assess qualitative study designs. Whereas no specific risk of bias tool exists for the assessment of observational, cross-sectional study designs. In light of this, this review used the domain-based risk of bias evaluation tool developed by Hoy *et*.*al*[17] as a foundation. Although this tool considers risk of bias in prevalence studies, it acts as a template for consideration of the risk of bias in cross-sectional studies. Minor modifications were made to this tool and were guided by Draugalis *et*.*al*[18] and the *Strengthening the Reporting of Observational studies in Epidemiology* (STROBE) statement.[19] The modified tool considered the risk of bias in observational cross-sectional studies and contained eleven items covering four domains of bias: selection bias, measurement bias, non-response bias, and bias of data analysis. The risk of bias for each domain was considered against pre-specified criteria (S1 File).

The findings of this review have been reported using the Preferred Reporting Items for Systematic Reviews and Meta-Analyses (PRISMA) guidelines.[20] Due to substantial heterogeneity in target populations, interjurisdictional policies, primary research aims, and data collection methods, the gathered results were not combined for pooled analysis. This review was registered with PROSPERO (ID: CRD42017067318).

## Results

### Study selection and characteristics

**Fig 1:**
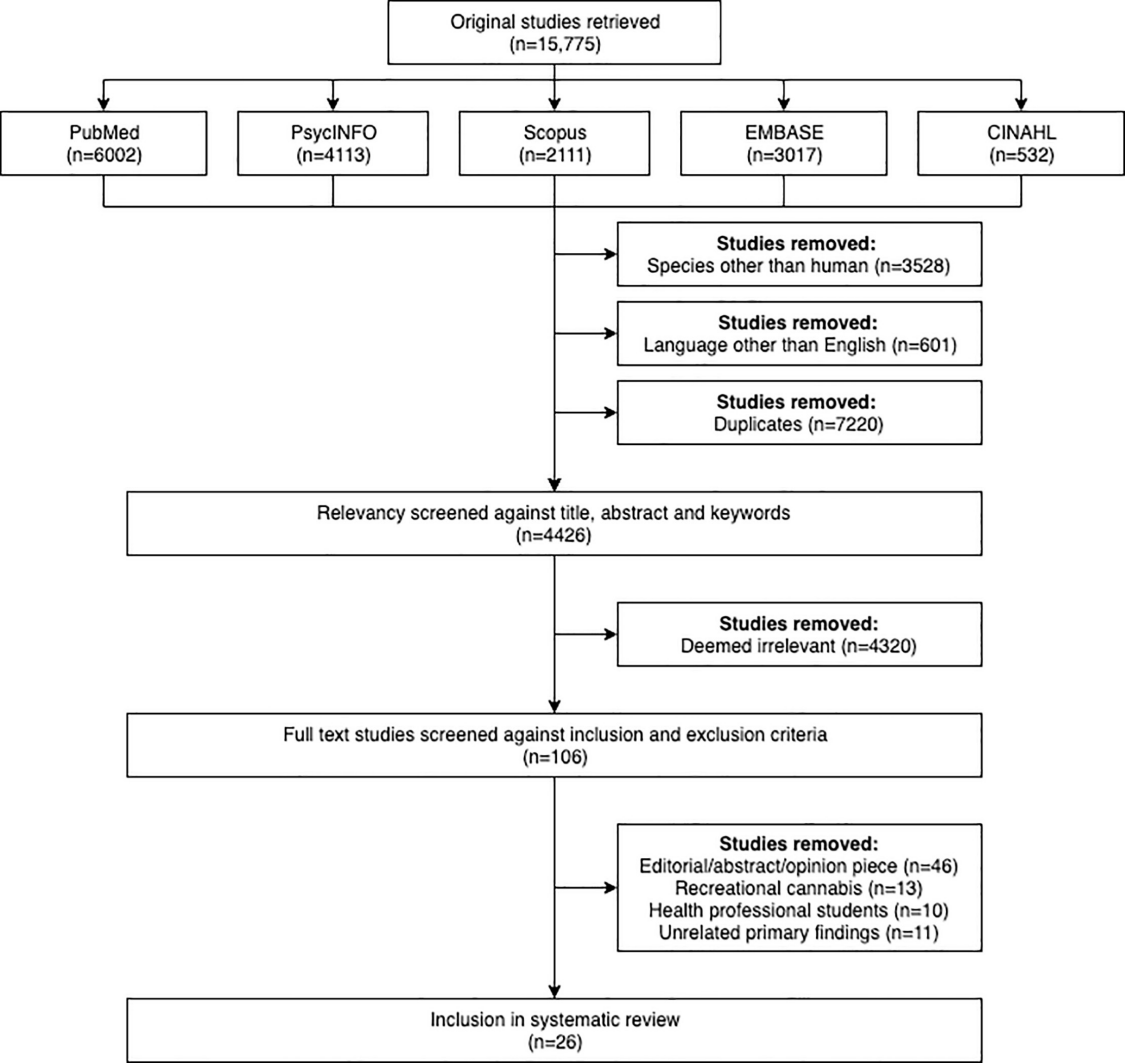
PRISMA flow diagram of included studies.

There were 15,775 studies retrieved through the search of the listed databases. From this total, 3528 were removed due to a focus on a species other than humans, 601 were removed due to being in a language other than English, and 7220 were removed as they were retrieved in duplicate. Furthermore, 4320 studies were removed as these were deemed to be irrelevant in the preliminary screening of the title, abstract, and keywords. This left 106 full-text studies for screening against inclusion and exclusion criteria. Eighty studies did not meet these pre-specified criteria, leaving twenty-six studies for inclusion in this systematic review.[21–46] A flow diagram of study selection is shown in Fig 1. Of the 26 studies included, 13 were conducted in the United States (US),[22, 25–28, 30, 33, 37, 38, 40–43] four were conducted in Canada,[23, 32, 39, 45] three were conducted in Australia[34–36] and Israel,[21, 31, 46] two were conducted in Ireland,[29, 44] and one covered an international sample.[24] There were 18 studies that sampled medical practitioners,[21, 24–26, 28–32, 34, 36, 37, 40–42, 44–46] and three studies that sampled pharmacists.[33, 35, 39] An additional five studies included in this review were grouped according to the high proportion of nursing professionals sampled.[22, 23, 27, 38, 43] One study sampled exclusively nurse practitioners[23] while the other four studies sampled a mixed cohort of medical and allied health professionals of which nurses were the predominant population.[22, 27, 38, 43] Altogether, these five studies had a pooled sample of 1,584 participants including 847 (53%) registered nurses and nurse practitioners, 495 (31%) medical practitioners, 118 (8%) pharmacists, 37 (2%) social workers, 21 (1.5%) physician assistants, and three (0.5%) osteopathic physicians. Also included in the pooled sample were 63 (4%) individuals described as ‘other’ which included an unknown proportion of medical practitioners and pharmacists. A summary of study characteristics is displayed in S2 Table.

### Risk of bias

**Fig 2:**
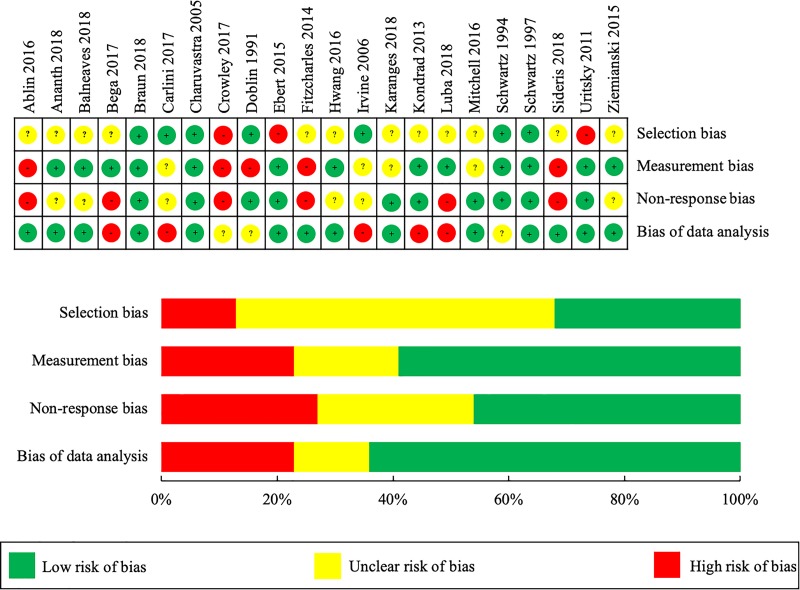
Risk of bias summary and graph–quantitative studies.

**Fig 3:**
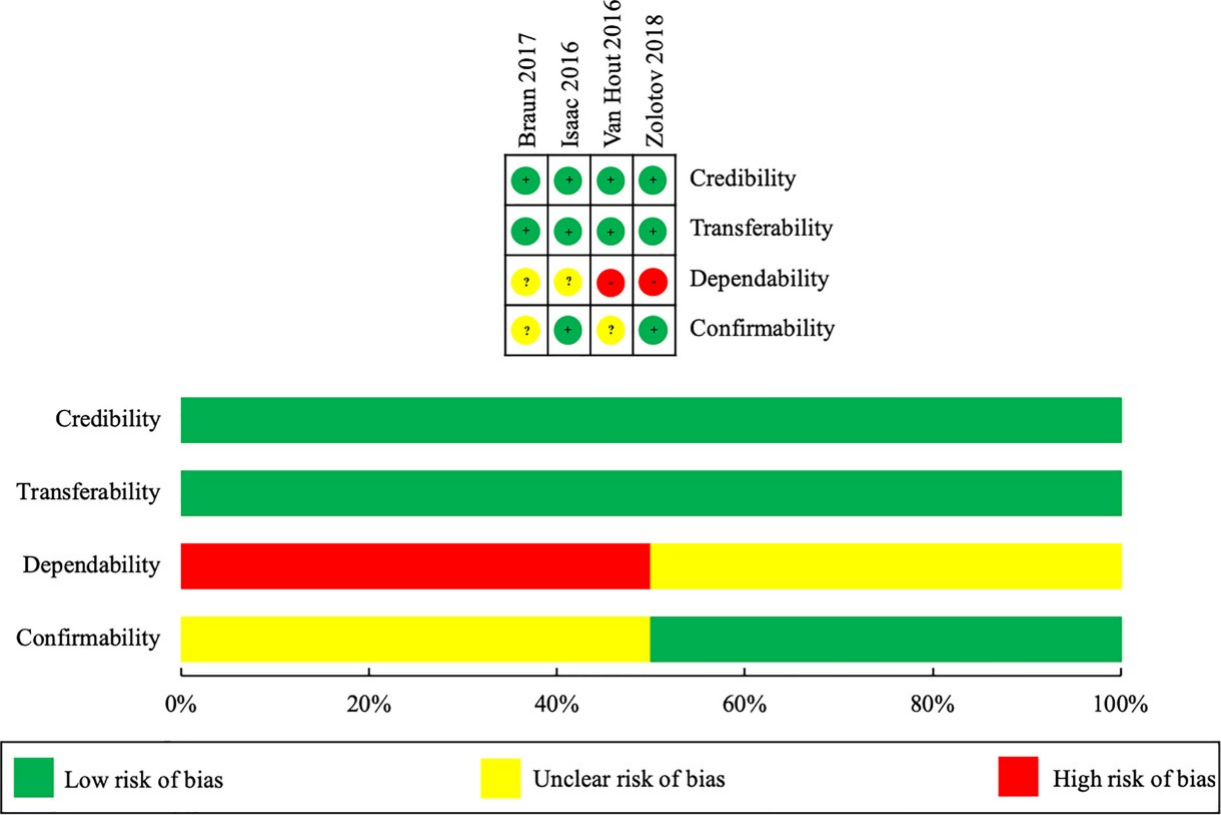
Risk of bias summary and graph–qualitative studies.

Of the quantitative studies, two were considered to have a low risk of bias for each domain.[25, 41] Although the remaining twenty studies had varying levels of potential bias present, the overall risk of bias across all included studies was low (Fig 2). This was especially true with respect to measurement bias, non-response bias, and bias of data analysis. The impact of selection bias was largely unclear, however. This was mainly due to many studies not clearly justifying their how their sample was representative of the study population, as well as the wide use of non-probability sampling. As the four qualitative studies differed in their philosophical paradigms, the ten-item CASP criteria framework was broken down and used to evaluate the main hallmarks of rigorous qualitative research: credibility, transferability, dependability, and confirmability. Credibility is the confidence that can be placed in the truth of the research findings while transferability is the degree by which results can be transferred to other contexts. Dependability involves an evaluation of the findings and interpretations such that results are supported by the data received and confirmability questions whether the results are shaped by the participants or other potential biases. Within their respective philosophical paradigms, the potential influence of bias for the qualitative studies was considered minimal (Fig 3).

### Beliefs, knowledge and concerns

#### Medical practitioners

Of the 18 studies investigating medical practitioners’ perspectives, none presented results that outright rejected the clinical usefulness of medicinal cannabis. Six studies reported the majority of participants supporting the clinical usefulness of medicinal cannabis.[21, 29, 31, 34, 36, 45] A further five studies indicated that participants believed medicinal cannabis to be a viable therapeutic option.[25, 26, 30, 42, 46] In contrast, three studies revealed that the majority of participants believed medicinal cannabis to have a limited role in their respective fields[32, 37, 41] while a further study reported a lack of confidence in the use of cannabis as a treatment option.[40] In addition to the support and opposition of medicinal cannabis, three studies reported a high proportion of neutrality when participants were asked whether medicinal cannabis had any clinical utility.[24, 28, 44] Regardless of pre-conceived beliefs; however, the relative support for medicinal cannabis increased if all other treatment options were exhausted or if therapy was to be used in the palliative domain.[26, 28, 29, 36, 37, 40, 41]

With respect to knowledge, seven studies reported the self-perceived knowledge of the pharmacology of phyto–and synthetocannabinoids to be predominantly very low on a five-point Likert scale.[21, 31, 32, 34, 36, 37, 42, 45] Similarly, five of these studies also demonstrated that self-perceived legislative and procedural knowledge, including the specifics around prescribing, cultivation and distribution was very low.[21, 32, 36, 42, 45] When discussing education, three studies reported a desire for more formal education across undergraduate, postgraduate, and professional development curricula.[24, 31, 37] Furthermore, 70% of Canadian physicians stated that they would feel more comfortable authorising medicinal cannabis if formal education was provided.[45] Collectively, a self-perceived lack of knowledge was reported as a significant barrier to authorisation, yet a lack of easy-to-access information was also reported to be an noteworthy barrier.[26, 36, 42, 45] Additionally, two studies reported that 20% and 60% of their respective samples used the news/media as a resource to inform clinical decision-making.[24, 37]

Medical practitioners raised issues surrounding self-cultivation such as reproducibility and contamination, the potential for recreational misuse, the potential for diversion into illicit channels, the risk of drug-drug interactions, and driving under the influence of cannabis.[21, 24, 32, 36, 37, 42, 44, 45] Furthermore, medical practitioners were mindful of the potential risks associated with uncontrolled supply.[31, 37] Finally, concern for the health of individual patients was mostly focused around psychiatric adverse effects. Three studies indicated that medical practitioners believed negative mental health outcomes were positively correlated with the use of medicinal cannabis.[25, 26, 29, 37]

#### Pharmacists

Of the three studies sampling pharmacists, only one captured pharmacists’ beliefs. Mitchell *et*.*al*[39] reported that 55% of Canadian hospital pharmacists agreed that overall, medicinal cannabis was effective. Beliefs were not reported in relation to a specific indication.

Overall, pharmacists’ self-reported knowledge was quite low.[33, 35, 39] Hwang *et*.*al*[33] reported that pharmacists considered themselves to be lacking knowledge surrounding the pharmacology, pharmacokinetics and pharmacodynamics of medicinal cannabis. Additionally, legislative and procedural knowledge around prescribing, cultivation and distribution was also lacking for many pharmacists.[33, 39] With respect to education, 66% and 65% of Canadian hospital pharmacists reported receiving no undergraduate or professional development respectively on medicinal cannabis.[39] There was a self-reported desire from sampled Minnesotan and Australian pharmacists for further education.[33, 35] Finally, pharmacists stated that a lack of accessible and robust literature was a major limitation such that when faced with a clinical question, the majority of pharmacists used self-directed online learning to inform practice.[39]

Pharmacists reported a number of concerns surrounding a lack of quality controlled formulations, the potential for recreational misuse, the possibility for diversion into illicit channels, psychiatric co-morbidities, and the risk of drug-drug interactions.[33, 35, 39] When considering overall patient safety, Mitchell *et*.*al*[39] reported that 55% of Canadian hospital pharmacists either disagreed or were unsure whether medicinal cannabis was safe. Moreover, Hwang *et*.*al*[33] reported that 55% of participating pharmacists felt uncomfortable with the potential link between medicinal cannabis and psychiatric illness.[33]

#### Nurses combined with other medical and allied health professionals

Participants within this pooled sample were largely supportive of medicinal cannabis and although not all supported the use of medicinal cannabis, very few rejected its clinical utility. Conditional to the survey questions, these five studies reported: (a) a willingness to help children with cancer access medicinal cannabis[22], (b) ‘turning a blind eye’ to the unlawful use of medicinal cannabis because of a belief in its benefits[43], (c) the belief that medicinal cannabis is useful for chronic conditions[27], (d) support for the clinical utility of medicinal cannabis[38] and (e) comfort supporting a patient’s right to access medicinal cannabis.[23]

Self-perceived knowledge relating to the endocannabinoid system and the pharmacology of cannabinoids was largely considered poor.[23, 27] Knowledge surrounding procedural aspects of medicinal cannabis therapy such as dosing, formulation differences, and legislation surrounding access, distribution, and supply was also poor.[22, 23, 27] For many participants, a major barrier to engagement was a self-perceived lack of knowledge and education surrounding medicinal cannabis.[22, 23, 27, 43] Two studies indicated a strong desire for additional education.[23, 27] Moreover, Balneaves *et*.*al*[23] reported that 90% of participants stated they would feel more comfortable engaging with medicinal cannabis after more education. Carlini et.al[27] reported the use of news/media and patient experience to inform clinical decisions where formal education was lacking.[27]

The predominant concerns encountered by this pooled sample were the potential for recreational misuse, psychiatric co-morbidities, the absence of robust clinical guidelines, and the risk of drug-drug interactions.[22, 23, 27] Additional concerns were raised surrounding the risks associated with uncontrolled supply.[43] Three studies reported the side effect profile of medicinal cannabis to be a considerable reason for therapy avoidance.[23, 38, 43] Whereas, Carlini *et*.*al*[27] reported that participants believed serious mental health deterioration could eventuate from prescriber-initiated medicinal cannabis therapy. This was thought to be a significant limitation to its clinical applicability.[27]

## Discussion

### Principle findings

This systematic review examined health professionals’ beliefs, knowledge and concerns surrounding the delivery medicinal cannabis. The three research questions will now be discussed.

*(1) How do health professionals feel about the use of medicinal cannabis in clinical practice*?

Defining how health professionals felt about medicinal cannabis lacked consistency across the included studies. The notion of support for medicinal cannabis was measured using a number of methods. These included the belief in a prescriber’s right to prescribe medicinal cannabis, the belief in a patient’s right to obtain medicinal cannabis, and support for the clinical usefulness of medicinal cannabis. Given such a high level of heterogeneity, it is difficult to consolidate these responses to offer specific answers. More broadly, however, health professionals were relatively supportive of the use of medicinal cannabis in clinical practice. This notion was consistent across the three predominant professional populations of medicine, pharmacy, and nursing. It is worthwhile commenting, however, that although there was a general impression of support, this did vary according to publication year, geographical location, and medical speciality. Primarily, older studies presented health professionals’ views that were generally less supportive of medicinal cannabis than more recently published studies. Among other factors, this presents questions around the impact of social influence upon health professional beliefs. Secondly, support for medicinal cannabis varied between jurisdictions with no obvious patterns relative to jurisdictional leniency or strictness. These results highlight that the influence of an individual’s professional environment perhaps needs further investigation. Finally, health professionals were more likely to show signs of support if they were in a speciality field compared with those in general or community practice. This perhaps highlights the importance of environmental context, but more specifically the importance of one’s self-perceived professional role and identity. Yet, it is important to recognise that there is a fundamental difference between being supportive of medicinal cannabis and being directly responsible for its delivery. As such, support for medicinal cannabis should not be seen as willingness to deliver. More research is required to develop a link between support and willingness.

*(2) How knowledgeable are health professionals regarding medicinal cannabis*?

Knowledge was broken down across legislative, and clinical fields. Legislative knowledge included cultivation, distribution, and patient acquisition. Clinical knowledge included aspects such as pharmacology, pharmacokinetics, and pharmacodynamics of medicinal cannabis as well as efficacy, dosing, and adverse drug reactions. There was a degree of consistency in the way these questions were asked with most utilising Likert scales. Yet, the studies included in this review all measured self-perceived knowledge. Thus, these results do not necessarily offer a true representation of knowledge, but rather a perception of knowledge that is perhaps influenced by external factors. Self-perceived knowledge was deemed poor across legislative and clinical fields and this was consistent across medicine, pharmacy, and nursing. In addition to a universal lack of self-perceived knowledge relating to medicinal cannabis, many health professionals highlighted the need for further education and easy-to-access information. Where health professionals deemed evidence-based resources inaccessible, other forms of evidence where being used to generate knowledge. These included self-directed online learning, the news/media, and past patient experiences. Interestingly, for many health professionals, support for medicinal cannabis was reinforced by beliefs about clinical usefulness. Yet, these same participants often reported having a self-perceived lack of clinical knowledge. It is then interesting to question whether beliefs surrounding the benefits of medicinal cannabis are grounded in evidence or are an artefact of something else.

*(3) What concerns exist for health professionals regarding the delivery of medicinal cannabis*?

The concerns of health professionals relating to the delivery of medicinal cannabis were explored using two methods: (a) participants were asked to comment on what their concerns were, or (b) five-point Likert scales were used to quantify the level of concern for pre-specified items. Where the former allowed participants to outline what concerns they had, the latter tended to identify the level of concern for pre-specified concepts. Nevertheless, there was a high degree of consistency between the concerns that emerged from free-text responses and those that were assessed using Likert scales. As with both the previous research questions, there were no major differences across medicine, pharmacy, and nursing. Health professional concerns were divided into two major categories–concerns for direct patient harm and concern for indirect societal harm. Health professionals were mostly concerned with the risk of psychiatric adverse drug reactions while generally understating the risks of other adverse drug reactions. Furthermore, health professionals consistently acknowledged that patients requiring medicinal cannabis are often those with significant co-morbidities and polypharmacy, and hence expressed concern regarding the potential for drug-drug interactions. A number of indirect public health concerns emerged as being problematic, but none more than the fear that cannabis would be obtained ‘medicinally’ as a legal façade for recreational use. As above, it is interesting to view these concerns within the context of a lack of knowledge. While it is hard to identify whether health professionals are basing their beliefs of direct and indirect consequences on the results of peer-reviewed literature or the negative stigma associated with cannabis, what is evident is that health professionals need more guidance.

### Implications for research

An interesting additional finding and a limitation to the overall completeness of this review was a lack of theoretical framing when exploring the factors that underpin the delivery of medicinal cannabis. It is important to recognise that due to the implicit common-sense models of behaviour applied, these results are limited to the beliefs, knowledge, and concerns of health professionals.[47, 48] Not all possible variables have been examined and as such, these results only convey a partial description of the target behaviour–in this case the, delivery of medicinal cannabis. Yet, there are attempts to translate these results into intervention strategies without fully understanding the nature of the target behaviour in context. For example, Fitzcharles *et*.*al*[32] asked participants about their medicinal cannabis beliefs and knowledge. The authors concluded that Canadian rheumatologists lacked confidence and competence and given this uncertainty, participating rheumatologists reported a resistance to authorisation. The authors suggest additional guidance for the authorisation of medicinal cannabis in the treatment of rheumatic conditions as a potential intervention to lessen the resistance identified. The authors provide no consideration for other potentially important variables such as emotional processing, social influence, behavioural regulation, or reinforcement.[49] Without considering the complete nature of a given behaviour in an appropriate context, any proposed intervention strategy will seldom be complete or grounded in evidence.

### Strengths and limitations

This study was reported according to PRISMA. Furthermore, a pre-specified study protocol was used which identified all studies discussing health professionals within the context of delivering medicinal cannabis. The full-text review, data extraction, and bias assessment were performed by two independent researchers whilst inclusion and exclusion criteria were explicit and applied uniformly. Nevertheless, unpublished studies or studies indexed in smaller databases could have been missed. Additionally, studies published in a language other than English will also have been missed. Furthermore, articles indexed as either an abstract, editorial or opinion piece were intentionally excluded. This decision was made due to the inability to adequately assess methodological quality and perform critical appraisal.

The modified risk of bias tool used to assess the quantitative studies needs to be used within the context of its limitations. Although modifications were made according to explicit criteria for the development and reporting of observational, cross-sectional studies, the original tool was developed for prevalence studies. It was deemed that prevalence studies represented a closer philosophical resemblance to observational, cross-sectional studies than did intervention studies which is why more established scales such as the Newcastle-Ottawa-Scale and the Downs and Black checklist were not used. Overall, the probability of bias influencing these results is considered to be small; however, the potential for publication bias cannot be completely ignored. It is unlikely that these results will directly inform clinical practice, but rather the intent is to inform further research exploring the factors that underpin the delivery of medicinal cannabis.

## Conclusion

Through an analysis of the available literature, this systematic review identified three major points. Primarily, health professionals are relatively supportive of medicinal cannabis in clinical practice, yet this support is often counterbalanced by a lack of confidence, a lack of self-reported competence, and concerns for the associated risks. Secondly, there was universal self-reported lack of knowledge regarding legislative and clinical domains. It is unclear what impact potentially biased information sources are having on the acquisition of knowledge particularly where robust sources are scarce. Finally, the most concerning direct patient harm of medicinal cannabis was the risk of psychiatric adverse effects. The most concerning indirect societal harm was the potential for recreational misuse of medically acquired cannabis. The comprehensiveness of these results is limited by the use of implicit common-sense models of behaviour. Before strategies can be are implemented to change health professional behaviour, a more comprehensive description of the factors that underpin the delivery of medicinal cannabis must be sought. Only then can strategies grounded in evidence, be implemented.

## Supporting information

S1 Table Database searching phrases. (DOCX)

S2 Table Study characteristics and summary of findings for included studies. (DOCX)

S3 Table PRISMA checklist. (DOCX)

S1 File The eleven-item modified domain-based risk of bias assessment tool and pre-specified risk of bias criteria. (PDF)

## References

[1] Li H-L. An archaeological and historical account of cannabis in China. Econ Bot. 1973;28(4):437–48. 10.1007/bf02862859

[2] Zuardi AW. History of cannabis as a medicine: A review. Rev Bras Psiquiatr. 2006;28(2):153–7. doi: /S1516-44462006000200015 16810401 10.1590/s1516-44462006000200015

[3] United States Pharmacopeial Convention. United States Pharmacopeia. 3rd ed Philadelphia, PA: Lippincott, Grambo & Co.; 1851.

[4] Robinson V. An Essay on hasheesh including observations and experiments. Am J Psychiatry. 1912;70(1):276–7. 10.1176/ajp.70.1.276

[5] UN General Assembly. The 1972 protocol amending the 1961 Single Convention on Narcotic Drugs, 1975 Available from: https://www.incb.org/documents/Narcotic-Drugs/1961-Convention/convention_1961_en.pdf. 4486197

[6] Devane WA, Hanus L, Breuer A, Pertwee RG, Stevenson LA, Griffin G, et al Isolation and structure of a brain constituent that binds to the cannabinoid receptor. Science. 1992;258(5090):1946–9. 1470919 10.1126/science.1470919

[7] Howlett A, Champion T, Wilken G, Mechoulam R. Stereochemical effects of 11-OH-Δ8-tetrahydrocannabinol-dimethylheptyl to inhibit adenylate cyclase and bind to the cannabinoid receptor. Neuropharmacology. 1990;29(2):161–5. 2158635 10.1016/0028-3908(90)90056-w

[8] Little P, Compton D, Johnson M, Melvin L, Martin B. Pharmacology and stereoselectivity of structurally novel cannabinoids in mice. J Pharmacol Exp Ther. 1988;247(3):1046–51. 2849657

[9] Barnes M, Barnes J. Cannabis: the evidence for medical use, 2016. Available from: http://www.drugsandalcohol.ie/26086/1/Cannabis_medical_use_evidence.pdf.

[10] Crane NA, Schuster RM, Fusar-Poli P, Gonzalez R. Effects of cannabis on neurocognitive functioning: Recent advances, neurodevelopmental influences, and sex differences. Neuropsychol Rev. 2013;23(2):117–37. 10.1007/s11065-012-9222-1 23129391 PMC3593817

[11] Nutt D, King LA, Saulsbury W, Blakemore C. Development of a rational scale to assess the harm of drugs of potential misuse. Lancet. 2007;369(9566):1047–53. 10.1016/S0140-6736(07)60464-4 17382831

[12] Whiting PF, Wolff RF, Deshpande S, Di Nisio M, Duffy S, Hernandez AV, et al Cannabinoids for medical use: A systematic review and meta-analysis. Jama. 2015;313(24):2456–73. 10.1001/jama.2015.6358 26103030

[13] Proposition 215, California Department of Public Health (1996).

[14] Hazekamp A, Ware MA, Muller-Vahl KR, Abrams D, Grotenhermen F. The medicinal use of cannabis and cannabinoids–An international cross-sectional survey on administration forms. J Psychoactive Drugs. 2013;45(3):199–210. 10.1080/02791072.2013.805976 24175484

[15] Belackova V, Ritter A, Shanahan M, Chalmers J, Hughes C, Barratt M, et al Medicinal cannabis in Australia–Framing the regulatory options. Sydney: Drug Policy Modelling Program; 2015.

[16] Chapman SA, Spetz J, Lin J, Chan K, Schmidt LA. Capturing heterogeneity in medical marijuana policies: A taxonomy of regulatory regimes across the United States. Subst Use Misuse. 2016;51(9):1174–84. 10.3109/10826084.2016.1160932 27191472 PMC4979574

[17] Hoy D, Brooks P, Woolf A, Blyth F, March L, Bain C, et al Assessing risk of bias in prevalence studies: Modification of an existing tool and evidence of interrater agreement. J Clin Epidemiol. 2012;65(9):934–9. 10.1016/j.jclinepi.2011.11.014 22742910

[18] Draugalis JR, Coons SJ, Plaza CM. Best practices for survey research reports: a synopsis for authors and reviewers. Am J Pharm Educ. 2008;72(1):11 18322573 10.5688/aj720111 PMC2254236

[19] Strengthening the reporting of observational studies in epidemiology (STROBE) statement: guidelines for reporting observational studies. BMJ. 2007;335(7626). 10.1136/bmj.39386.490150.94 PMC2034723 17947786

[20] Moher D, Liberati A, Tetzlaff J, Altman DG. Preferred reporting items for systematic reviews and meta-analyses: The PRISMA statement. Ann Intern Med. 2009;151(4):264–9. 19622511 10.7326/0003-4819-151-4-200908180-00135

[21] Ablin JN, Elkayam O, Fitzcharles MA. Attitudes of israeli rheumatologists to the use of medical cannabis as therapy for rheumatic disorders. Rambam Maimonides Med J. 2016;7(2). 10.5041/rmmj.10239 27101219 PMC4839539

[22] Ananth P, Ma C, Al-Sayegh H, Kroon L, Klein V, Wharton C, et al Provider perspectives on use of medical marijuana in children with cancer. Pediatrics. 2018;141(1). 10.1542/peds.2017-0559 29233937 PMC5744275

[23] Balneaves LG, Alraja A, Ziemianski D, McCuaig F, Ware M. A national needs assessment of canadian nurse practitioners regarding cannabis for therapeutic purposes. Cannabis Cannabinoid Res. 2018;3(1):66–73. 10.1089/can.2018.0002 29588917 PMC5868330

[24] Bega D, Simuni T, Okun MS, Chen X, Schmidt P. Medicinal cannabis for Parkinson's disease: practices, beliefs, and attitudes among providers at the national Parkinson Foundation Centers of Excellence. Mov Disord Clin Pract. 2017;4(1):90–5. 10.1002/mdc3.12359 30713951 PMC6353502

[25] Braun IM, Meyer FL, Gagne JJ, Nabati L, Yuppa DP, Carmona MA, et al Experts' perspectives on the role of medical marijuana in oncology: A semistructured interview study. Psychooncology. 2017 10.1002/pon.4365 28040884

[26] Braun IM, Wright A, Peteet J, Meyer FL, Yuppa DP, Bolcic-Jankovic D, et al Medical oncologists' beliefs, practices, and knowledge regarding marijuana used therapeutically: A nationally representative survey study. J Clin Oncol. 2018;36(19):1957–62. 10.1200/JCO.2017.76.1221 29746226 PMC6553839

[27] Carlini BH, Garrett SB, Carter GT. Medicinal cannabis: a survey among health care providers in washington state. AM J Hosp Palliat Med. 2017;34(1):85–91. 10.1177/1049909115604669 26377551

[28] Charuvastra A, Friedmann PD, Stein MD. Physician attitudes regarding the prescription of medical marijuana. J Addict Dis. 2005;24(3):87–93. 10.1300/J069v24n03_07 16186085

[29] Crowley D, Collins C, Delargy I, Laird E, Van Hout MC. Irish general practitioner attitudes toward decriminalisation and medical use of cannabis: Results from a national survey. Harm Reduct J. 2017;14(1). 10.1186/s12954-016-0129-7 28086792 PMC5237358

[30] Doblin RE, Kleiman MA. Marijuana as antiemetic medicine: a survey of oncologists' experiences and attitudes. J Clin Oncol. 1991;9(7):1314–9. 10.1200/JCO.1991.9.7.1314 2045870

[31] Ebert T, Zolotov Y, Eliav S, Ginzburg O, Shapira I, Magnezi R. Assessment of israeli physicians’ knowledge, experience and attitudes towards medical cannabis: A pilot study. Isr Med Assoc J. 2015;17(7):437–41. 26357721

[32] Fitzcharles MA, Ste-Marie PA, Clauw DJ, Jamal S, Karsh J, LeClercq S, et al Rheumatologists lack confidence in their knowledge of cannabinoids pertaining to the management of rheumatic complaints. BMC Musculoskelet Disord. 2014;15:258 10.1186/1471-2474-15-258 25080153 PMC4121007

[33] Hwang J, Arneson T. Minnesota pharmacists and medical cannabis: A survey of knowledge, concerns, and interest prior to program launch. Pharm Ther. 2016;41(11):716–22. PMC5083080 27904305

[34] Irvine G. Rural doctors' attitudes to and knowledge of medicinal cannabis. J Law Med. 2006;14(1):135–42. 16937787

[35] Isaac S, Saini B, Chaar BB. The role of medicinal cannabis in clinical therapy: Pharmacists' perspectives. PLoS ONE. 2016;11(5). 10.1371/journal.pone.0155113 27171490 PMC4865212

[36] Karanges EA, Suraev A, Elias N, Manocha R, McGregor IS. Knowledge and attitudes of Australian general practitioners towards medicinal cannabis: A cross-sectional survey. BMJ Open. 2018;8(7). 10.1136/bmjopen-2018-022101 29970456 PMC6042562

[37] Kondrad E, Reid A. Colorado family physicians' attitudes toward medical marijuana. J Am Board Fam Med. 2013;26(1):52–60. 10.3122/jabfm.2013.01.120089 23288281

[38] Luba R, Earleywine M, Farmer S, Slavin M. Cannabis in end-of-life care: Examining attitudes and practices of palliative care providers. J Psychoactive Drugs. 2018:1–7. 10.1080/02791072.2018.1462543 29714640

[39] Mitchell F, Gould O, LeBlanc M, Manuel L. Opinions of hospital pharmacists in canada regarding marijuana for medical purposes. Can J Hosp Pharm. 2016;69(2):122–30. 27168633 10.4212/cjhp.v69i2.1539 PMC4853179

[40] Schwartz RH, Beveridge RA. Marijuana as an antiemetic drug: How useful is it today? Opinions from clinical oncologists. J Addict Dis. 1994;13(1):53–65. 10.1300/J069v13n01_05 7503819

[41] Schwartz RH, Voth EA, Sheridan MJ. Marijuana to prevent nausea and vomiting in cancer patients: A survey of clinical oncologists. South Med J. 1997;90(2):167–72. 9042166 10.1097/00007611-199702000-00001

[42] Sideris A, Khan F, Boltunova A, Cuff G, Gharibo C, Doan LV. New York physicians' perspectives and knowledge of the state medical marijuana program. Cannabis Cannabinoid Res. 2018;3(1):74–84. 10.1089/can.2017.0046 29662957 PMC5899285

[43] Uritsky TJ, McPherson ML, Pradel F. Assessment of hospice health professionals' knowledge, views, and experience with medical marijuana. J Palliat Med. 2011;14(12):1291–5. 10.1089/jpm.2011.0113 22077541

[44] van Hout MC, Collins C, Delargy I, Crowley D. Irish General Practitioner perspectives toward decriminalisation, legalisation and cannabis for therapeutic purposes (CTP). Int J Ment Health Addict. 2016:1–14. 10.1007/s11469-016-9710-2

[45] Ziemianski D, Capler R, Tekanoff R, Lacasse A, Luconi F, Ware MA. Cannabis in medicine: A national educational needs assessment among Canadian physicians BMC Med Educ. 2015;15(1). 10.1186/s12909-015-0335-0 25888752 PMC4374299

[46] Zolotov Y, Vulfsons S, Zarhin D, Sznitman S. Medical cannabis: An oxymoron? Physicians' perceptions of medical cannabis. Int J Drug Policy. 2018;57:4–10. 10.1016/j.drugpo.2018.03.025 29653439

[47] Michie S, Fixsen D, Grimshaw JM, Eccles MP. Specifying and reporting complex behaviour change interventions: The need for a scientific method. Implement Sci. 2009;4:40 10.1186/1748-5908-4-40 19607700 PMC2717906

[48] Michie S, van Stralen MM, West R. The behaviour change wheel: A new method for characterising and designing behaviour change interventions. Implement Sci. 2011;6:42 10.1186/1748-5908-6-42 21513547 PMC3096582

[49] Atkins L, Francis J, Islam R, O'Connor D, Patey A, Ivers N, et al A guide to using the Theoretical Domains Framework of behaviour change to investigate implementation problems. Implement Sci. 2017;12(1):77 10.1186/s13012-017-0605-9 28637486 PMC5480145

