# Supplementary material for: Health professional beliefs, knowledge, and concerns surrounding medicinal cannabis – A systematic review
Source: PLoS One. 2019 May 6;14(5):e0216556. doi: 10.1371/journal.pone.0216556 (PMC6502454; doi:10.1371/journal.pone.0216556)
Supplement: S1 Table — (DOCX) [file pone.0216556.s001.docx]

S1 Table. Database searching phrases

| Database | Search phrase |
| --- | --- |
| Pubmed | ("Cannabinoids"[Mesh] OR "medical marijuana"[Mesh] OR ((medical[tiab] OR medicinal[tiab]) AND (cannabis[tiab] OR marijuana[tiab]))) AND (("Behavior and Behavior Mechanisms"[Mesh]) OR (attitud*[tiab] OR perce*[tiab] OR opinion*[tiab] OR belief*[tiab] OR knowl*[tiab])) |
| PsycINFO | ((medical OR medicinal) AND (cannabis OR marijuana)) OR cannabinoid) AND (attitude OR opinion OR perception OR belief OR view OR thought OR feeling OR behavior OR knowledge) |
| Scopus | (ti-ab-kw: ((medical OR medicinal) AND (cannabis OR marijuana))) AND (ti-ab-kw: attitude* OR perce* OR knowl* OR opinion* OR belie* OR feel* OR view* OR thought*) |
| EMBASE | (“medical cannabis”:de OR ((medical:ti,ab OR medicinal:ti,ab) AND (cannabis:ti,ab OR marijuana:ti,ab))) AND (behavior:de OR behaviour/syn OR perception/syn OR knowledge:ti,ab OR opinion:ti,ab OR belief:ti,ab OR view:ti,ab |
| CINAHL | ((medical OR medicinal) AND (cannabis OR marijuana OR cannabinoid) AND (attitudes or perceptions or opinions or thoughts or feelings or beliefs or behaviour or knowledge) |
