# Supplementary material for: Health professional beliefs, knowledge, and concerns surrounding medicinal cannabis – A systematic review
Source: PLoS One. 2019 May 6;14(5):e0216556. doi: 10.1371/journal.pone.0216556 (PMC6502454; doi:10.1371/journal.pone.0216556)
Supplement: S2 Table — (DOCX) [file pone.0216556.s002.docx]

S2 Table. Study characteristics and summary of findings for included studies

| **Article** | **Participants** | **Jurisdiction** | **Methods** | **Summary of findings** |
| --- | --- | --- | --- | --- |
| Ablin et.al; 2016 | 23 Rheumatologists | Israel | Cross-sectional survey | 17% believed cannabis had no therapeutic role; 9% no response; 74% expressed some role for cannabinoids for treatment of rheumatic disease. 82.6% were willing to prescribe if conventional treatment failed, while only 8.7% would consider prescribing on patient request irrespective of previous treatment experience. Confidence in prescribing: 78% not confident to prescribe; confidence in knowledge of dose, frequency & route of administration: 78% not confident & 13% somewhat confident. Major concerns included drug diversion; use for recreational purposes rather than medical; psychiatric co-morbidities; history of alcohol or drug addiction; drug interactions; driving; lack of clinical knowledge regarding exact mechanism of action of cannabinoids & unwanted effects on cognition, mood & sleep. |
| Ananth et.al; 2018 | 288 Paediatric oncology providers  (170 nurses; 83 physicians; 29 nurse practitioners; 3 social workers and 3 ‘other’) | US  [IL, MA and WA] | Cross-sectional survey | Most providers were willing to help children with cancer access MC (92%). Similarly, majority approved of the use of an oral MC formulation (89%). The vast majority reported that the most appropriate time to use MC near the end of life or as an adjunct with primarily palliative intent (89%). Overall, relatively good knowledge surrounding general legislative knowledge (whether state had legislation or not), yet, only 5% accurately identified state-specific legislation surrounding access, possession and cultivation. Majority of participants were not concerned about misuse potential nor where they concerned with prosecution for facilitating access. Most concern was the absence of standards around MC formulation, potency and dosing. |
| Balneaves et.al; 2018 | 182 Nurse practitioners | Canada | Cross-sectional survey | Although there were no overt attitudes whether NPs support the use of MC in clinical practice, 58.2% of NPs were comfortable supporting a patient’s access to MC. Overall, self-reported knowledge was considered poor. Particular gaps included: dosing and creating effective treatment plans, Canadian regulations, similarities and differences between formulations, mechanism of action and potential risks. There exists a high desire for more knowledge across the sample. The majority (90.7%) expressed that they would feel more comfortable discussing MC with patients if they had more education. There were a number of barriers identified, the most common included: lack of personal knowledge/education/information regarding MC, lack of clinical guidelines, possible drug-drug interactions, potential recreational diversion |
| Bega et.al; 2017 | 56 Neurologists | International | Cross-sectional survey | When considering the clinical usefulness of MC for their patients, 52% of neurologists took a neutral stance, 9% discouraged use while 39% supported use. Of the sample, only 10% had actively recommended using MC for symptoms of Parkinson’s. Albeit, the majority of neurologists believed MC would benefit their patient’s nausea, pain, appetite and anxiety while worsening balance, hallucinations, sleepiness and motivation. 93% of neurologists believed MC needed more emphasis in medical school curricula. When discussing where they obtain information 45% reported medical literature, 31% reported personal experience and 20% reported news/media. Neurologists felt MC would impact on patient’s memory, capacity to drive and were concerned about possible addiction. |
| Braun et.al; 2017 | 15 Oncology providers | US  [national] | Semi-structured interview | There was no outright negative response to medicinal cannabis use - perceived to be potentially beneficial in nausea, pain, some participants mentioned anorexia, cachexia, anxiety, sleep disturbance & coping; lack of quality research & therefore evidence base, mostly due to regulatory barriers. Providers stated a lack of quality research & therefore evidence base as a major barrier. Common concerns reported were: a lack of evidence-based knowledge; potential risk hospitals will prohibit medicinal cannabis on their premises (leading to abrupt discontinuation issues for hospitalised patients). Information was primarily being obtained via peer-reviewed literature and as anecdotal reports. The main safety considerations were: psychiatric issues including anxiety, dysphoria, paranoia, psychosis; cognitive impairment; driving dangers; infection; Some denied significant risks; while others considered cannabis safer than benzodiazepines and other narcotics. Participants were more concerned with psychiatric harms than the physical harms. |
| Braun et.al; 2018 | 237 Medical oncologists | US  [national] | Cross-sectional survey | 80% of participants reported having discussions about MC with patients and family, yet, the majority of these discussions were reported to be patient/family initiated. 46% reported recommending MC for cancer-related issues to at least one patients in the past year. When asked about the comparative effectiveness of MC, 34% of oncologists viewed MC as equal or more effective than standard pain treatment. When asked about utility as an adjunct to conventional pain treatment, 68% reported to a great/some extent. Additionally, 65% reported MC as more effective for cachexia, 48% as more effective for cancer-related nausea and vomiting, 45% for anxiety, 40% for general coping and 35% for sleep. Overall, only 30% of oncologists felt sufficiently knowledgeable regarding MC, yet, of those who had recommended MC in the past year, 56% didn’t consider themselves sufficiently knowledgeable. Although some participants were concerned with overdose and addiction with MC, when compared to opioids, the majority of participants ranked it as less of a concern. Comparatively, paranoia and confusion were considered comparable or higher than opioids while anxiety, depression and falls risk was equally mixed between comparable and less concerning. |
| Carlini et.al; 2017 | 494 Interdisciplinary health professionals  (205 nurse practitioners; 118 pharmacists; 72 nurses; 54 physicians; 21 physicians assistants; 21 ‘other’ and 3 osteopathic assistants) | US  [WA] | Cross-sectional survey | 73.7% believed cannabis can help patients with chronic, debilitating conditions; high level of support for legalising cannabis for medicinal purposes; pain was most frequent condition reported for prescribing. Participants generally had poor understanding of how cannabis worked & limited knowledge of endocannabinoid system; 64% reported limited knowledge of available products & where to get them; 62% were uncertain of dosing. There was an overall lack of knowledge regarding route of administration, side effects, interactions and mechanism of action. 77%, 87.2% and 96.1% believed more education was required in undergraduate, postgraduate and continuing development. 86% believed there should be an education mandate for prescribing. There was concern regarding the stigma associated with recreational cannabis use, limited clinical evidence of effectiveness and need for monitoring; narrow therapeutic window. Clinicians who had never prescribed cannabis were more likely to work in a clinic that prohibited them from doing so. Information was obtained via news & media, followed by patients, other clinicians & medical journals. 51.8% believed risk of abuse/dependence. |
| Charuvastra et.al; 2005 | 960 Specialist medical practitioners | US  [national] | Cross-sectional survey | Doctors should be able to legally prescribe cannabis as a medical therapy: 36% agreed; 26% were neutral & 37.8% disagreed; General interns & ObGyn more likely to support use. Variables such as age, gender & past substance misuse not influencers of approval of medicinal cannabis. The personal attitudes of medical practitioners towards substance misuse appeared to influence views of medicinal cannabis. |
| Crowley et.al; 2017 | 565 General practitioners | Ireland | Cross-sectional survey | 59% of the target population agree that cannabis should be legalised for medical purposes; 63%, 62% and 68% believe cannabis is effective for pain, MS and palliative care, respectively. Participants with higher level of training/experience in treatment of patients with opioid dependence more supportive of decriminalisation of cannabis. The age of prescriber (older less likely to support); gender (women less likely to support MC) and lack of advanced addiction training were influential. The perceived adverse effects of cannabis use: 82.7% - mental health; 60% - physical health; 77.3% linked use in young people with schizophrenia; diversion to illicit use; legalisation for medicinal purposes would increase use on population level. |
| Doblin et.al; 1991 | 1035 Oncologists | US  [national] | Cross-sectional survey | 54% of participants supported rescheduling of cannabis (to allow prescribing); 44% had recommended cannabis to at least one patient; older oncologists (graduated 50s) less likely to recommend cannabis compared with younger graduates; of the 28% of participants who believed they had sufficient information to compare cannabis with Marinol, a statistically significant proportion thought cannabis more effective & equally as safe as Marinol; 63% believed cannabis was efficacious in treatment of emesis; 64% reported that 50% or more of their patients experienced net benefits from cannabis; 44% believe cannabis to be both safe & efficacious; participants reported cannabis may be more useful in younger or in more cannabis-experienced patients; smoking better because 1) it enabled patient to self-titrate therapeutic dose 2) 'crude' cannabis has more active ingredients & 3) absorption of THC more reliable through lungs than gut. Major concerns included: treating small no. of pts (<150 pts/year); age of oncologist; lack of information, 30% felt they needed more information before prescribing medicinal cannabis; cannabis not legal - recommend patient smoke cannabis constitutes a criminal act. 44% believed cannabis to be safe & efficacious for treatment of emesis. |
| Ebert et.al; 2015 | 72 Specialist medical practitioners | Israel | Cross-sectional survey | 79.2% agreed treatment was helpful in chronic & terminally ill patients; 75% believed cannabis is a potential therapeutic agent; 75.8% believed cannabis is legitimate therapeutic agent. There were no significant differences across specialties and authors reported general acceptance particularly for treatment of cancer & terminal diseases. 80.2% rated themselves with medium-high to high knowledge of pharmacology & indications; 74.7% rated themselves with medium-high to high knowledge of risks & side effects while only 54.9% rated themselves with medium-high to high knowledge of doses & administration methods/routes. Previous experience as prescriber was a predictor of greater knowledge of doses/routes & risks/side effects. 88.8% felt more education should be available and 90.2% agreed that physicians certified to prescribe should undergo specific training to broaden their knowledge before certification. Barriers from patient perspective that physicians may suspect they are seeking cannabis for non-medicinal purposes |
| Fitzcharles et.al; 2014 | 128 Rheumatologists | Canada | Cross-sectional survey | 45% believe there is currently no role for any cannabinoid in rheumatic disease whereas 13% had previously recommended a trial. 60% would not currently recommend a trial of any cannabinoid product. 75% were not confident about current knowledge of cannabinoid molecules, 68% reported lack of confidence in current knowledge of endocannabinoid system in disease and 91% not confident to write prescription for cannabis indicating dose, frequency & route of administration. Concern was highlighted around the potential for drug overuse/misuse/diversion; mental health issues; working/operating machinery; concurrent medication use and cognitive impairment. 8.6% were unaware of any precautions to tell patients. |
| Hwang et.al; 2016 | 738 Pharmacists | US  [MN] | Cross-sectional survey | 77% believed (incorrectly) that a prescription was required to obtain cannabis; 71% did not know that advanced nurse practitioners have authority to certify patients and there was minimal knowledge around scheduling of cannabis. Majority rated themselves as having low cannabis knowledge - pharmacology (75.7%), kinetics (85.7%) & dynamics (84.3%) There was concern with a lack of targeted education; product storage & administration concerns regarding hospitalisation; psychoactive effects; potential for diversion & abuse; public & professional stigma. Most preferred to get their information from the State Board of Pharmacy and Health Department. |
| Irvine; 2006 | 32 General practitioners | Australia | Cross-sectional interview | 75% would consider prescribing where it was legal, supported by their peers & based on good quality evidence-based research. All would approve of government-sponsored clinical trials/research. Participants identified palliative care (81.3%); chronic pain (78.1%); AIDS-related wasting (59.4%); Chronic nausea (50%); MS (37.5%); Seizures (18.7%); Crohn's Disease (9.4%) as indications. There was concern with becoming overburdened by patients seeking cannabis once legal; lack of evidence for efficacy; need for GPs to receive training similar to that for methadone providers; identified need for uniform jurisdiction across Australia. Additional concern around risk of child access to liquid formulations. Participants happy for information to be either evidence-based or anecdote from patients. |
| Isaac et.al; 2016 | 34 Pharmacists | Australia | Semi-structured interview | The majority of participants expressed support & encouragement for use with a sense of duty of care to their patients. Many participants agreed that pharmacists should play a role in providing legitimate access. An extensive lack of knowledge was identified. The risks were not perceived as a barrier; supply through community pharmacies could increase security risk - increased risk of break-ins; acknowledged pharmacists' right to conscientious objection; lack of training & education & learning opportunities. Participants expressed concerns about potential risks associated with long-term use such as cognitive impairment and psychosis, however, most participants talked of weighing risks against benefits as with other drugs. |
| Karagnes et.al; 2018 | 640 General practitioners | Australia | Cross-sectional survey | 56.5% of participants reported that cannabis should be available on prescription for certain indications. Again, the majority (44%) believed they had patients who would benefit from therapy. Self-reported knowledge was considered poor across all dimensions. The vast majority disagreed with the statements: I know how to help my patients access MC, I understand the current regulatory approach, I have good knowledge surrounding MC, I am aware of different products/formulations, I understand the current RACGP position on MC. Impaired driving capacity, potential misuse and psychiatric adverse effects were perceived as some of the most concerned side effects, while almost 30% of participants stated they would not engage due to risk of misuse and dependence. The most common concern among participants was a perceived lack of knowledge and desire for more formal education. Other concerns included the need for more evidence, concerns about harms and cannabis seeking for recreational misuse. |
| Kondrad et.al; 2013 | 520 Family physicians | US  [CO] | Cross-sectional survey | 46% said physicians shouldn't recommend while 19% said they should. 27% reported there were physical health benefits to MC whilst 41% disagreed. 15% reported there were significant mental health benefits and 54% disagreed. Most were concerned with the lack of high-quality evidence of benefit. Additionally, there was concern about the potential harms. Participants obtained their information principally from medical literature, experiences with patients, the news media and other physicians. 61% considered there to be physical health risks & 64% believed there to be mental health risks. 76% agreed cannabis should be included in Colorado's Physician Drug Monitoring Program (secure, online database for tracking controlled drugs); concerns that majority of patients obtaining for recreational use (as legal protection) |
| Luba et.al; 2018 | Palliative care providers (58 nurses, 345 physicians, 23 ‘other’) | US | Cross-sectional survey | The majority supported the use of MC in palliative care (70%). These participants reported that MC was helpful for nausea (89.4%), appetite loss (89%) and pain (82%). Smaller reported MC as being helpful for sleep, irritability and emotional suffering. Many participants reported that they would recommend MC to patients with a terminal illness (61%). Reported concerns include low efficacy/symptoms management and adverse effects. |
| Mitchell et.al; 2016 | 769 Hospital pharmacists | Canada | Cross-sectional survey | 55.2% agreed cannabis is effective – age, education, area of clinical practice, province of work & personal experience was influential. Most thought cannabis was beneficial in AIDS-related anorexia & wasting syndrome, chronic pain, MS, oncology-related anorexia, nausea & vomiting. Most unsure if cannabis beneficial in treating glaucoma, inflammation & epilepsy. 66.7% reported an overall lack of knowledge. 65% reported no formal training while 54.8% had not read Canada's Cannabis for Medical Purposes Regulations. 12.1% had not heard of the regulation; 22.4% were aware of their institutions having a cannabis policy; 66% reported receiving no cannabis-related education in undergraduate degree; 64.5% reported receiving no formal training in continuing professional education. 48% reported not feeling comfortable to provide counselling to patients; 47.8% not comfortable providing advice to other health professionals; lack of knowledge & training, lack of evidence of efficacy; cannabis not a Health-Canada approved medication. Self-directed online learning major pathway for knowledge. |
| Schwartz et.al; 1997 | 1122 Oncologists | US  [national] | Cross-sectional survey | 13% believed that natural cannabis had better anti-emetic efficacy than pharmacological cannabinoids. 30% favoured legalisation of cannabis for medical reasons & of these, 50% also favoured legalisation for recreational use. There was concern with the lack of evidence of improved efficacy of smoked cannabis or pharmacological cannabinoids as anti-emetics compared with other drugs; in clinical trials, psychotropic effects of cannabis may mask true anti-emetic effects reported by participants; potential for abuse & illicit diversion, side effects impacting on quality of life - who is liable (state, doctor, pharmacist) if patient comes to harm. |
| Schwartz et.al; 1994 | 141 Oncologists | US  [national] | Cross-sectional survey | 18% had never prescribed cannabis & 46% had prescribed 10 times or less; participants estimated that pre- or post- chemo nausea & vomiting was reduced in 50% of patients who either smoked cannabis or used oral cannabis. It was noted that the side effects of ondansetron were less bothersome compared with short-term memory loss and issues operation of machinery with cannabis; 76% of participants reported that legalisation of natural cannabis would still not increase their prescribing of it. The side effects of cannabis were considered problematic. These included altered mental state, disturbance of fine motor coordination, anxiety, apprehension, light-headedness & syncope particularly in the elderly or cannabis-naïve patients. |
| Sideris et.al; 2018 | 164 Specialist physicians | US  [NY] | Cross-sectional survey | 71.2% of participants believed MC should be an option available for patients; however, 87% were not registered themselves to authorise MC. Of the sample, 73% were willing to refer patients to registered physicians as required. Main reasons for not registering themselves were their medical speciality and the federal status in the US. Other factors included prior experiences with patients, lack a medical literature for efficacy or safety and lack of knowledge. The majority self-rated their knowledge of the endocannabinoid system as generally poor while there was a high proportion of participants self-reporting a lack of knowledge surrounding the procedural requirements for patients and physicians. |
| Uritsky et.al; 2011 | 194 Hospice health care professionals  (131 nurses, 13 physicians, 34 pharmacists, 16 ‘other’) | US  [national] | Cross-sectional survey | 90% agreed with legalisation for cannabis for patients in palliative care and 86.1% agreed cannabis has medical benefits. Participants reported the following symptoms to be alleviated by cannabis: pain (87%), mood (55%), nausea & vomiting (2%), anorexia/appetite (36%). Participants were lacking knowledge about current indications and pharmacology, kinetics, dynamics and mode of action. Participants over 50yrs of age were significantly more likely than younger participants to disagree that smoked cannabis is addictive (64% vs 47%) - older participants may have more experience in palliative care & therefore less concerned with risk of addiction (or only responding in terms of the patient group they work with) |
| Van Hout et.al; 2016 | 565 General practitioners | Ireland | Content analysis | Prescribing under controlled, regulated conditions was perceived as being very different to legalising natural cannabis. Some were happy with prescribing but others not; some participants could perceive its potential benefits in chronic pain, palliative care & MS. Participants commented on lack of high-quality evidence to support use. Concern was raised regarding patient misuse; potential for illicit diversion - likened to opioids and benzodiazepines; weak evidence base & better forms of therapy are available; Further concern regarding side effects such as psychosis, however, many acknowledged that opioids & alcohol misuse are equally harmful; comments on the need for evidence-based approach to regulation & control |
| Ziemianski et.al; 2015 | 426 Medical practitioners | Canada | Cross-sectional survey | Participants believed those responsible for authorisation included specialists (85%), GPs (74%), nurse practitioners (25%). The perceived knowledge level relating to dosing & creating effective treatment plans for patients was poor and knowledge of similarities & differences between dried cannabis, and other forms of cannabis products was poor. The need for education was reported as strong or very strong by 64% (19% neutral & 19% not strong) - lack of education was felt to hinder discussions with patients & ability to treat patients. 65% concerned that patients requesting cannabis actually want it for recreational use (misuse & illicit diversion). Furthermore, the lack of evidence-based guidelines data on risks & benefits was concerning. 71% reported they would feel more comfortable discussing with patients/family members if they had more education. 70% reported that education would enable them to better treat patients. Concerns surrounding liability were raised.  Peer-reviewed literature reviews, online learning programs as part of CPD, other online resources, workshops and small learning sessions and symposia/conferences were highlighted as the preferred options for obtaining information. |
| Zolotov et.al; 2018 |  | Israel | Semi-structured interviews | Two major narratives were identified: ‘cannabis as a medicine’ and ‘cannabis as a non-medicine’. Although these narratives represented the polarities of physician perceptions, for many, these narratives coexisted, and physicians moved between them continuously. The ‘cannabis as a non-medicine’ narrative revolved around a lack of structured evidence, a lack of training and education, the concept of smoking as contradictory in health, misuse potential and diversion. Contrastingly, the ‘cannabis as a medicine’ narrative focused on anecdotal evidence and the willingness to overlook potential evidence deficits and harms in the palliative domain of care. Overall, health professionals were at a crossroads between their professional identity in upholding the ethical principles and biomedical standards of the medical professional and their role in aiding patients who are suffering. |
