# Supplementary material for: Health professional beliefs, knowledge, and concerns surrounding medicinal cannabis – A systematic review
Source: PLoS One. 2019 May 6;14(5):e0216556. doi: 10.1371/journal.pone.0216556 (PMC6502454; doi:10.1371/journal.pone.0216556)
Supplement: S1 File — (PDF) [file pone.0216556.s004.pdf]

S1 File. The eleven-item modified domain-based risk of bias assessment tool and pre-specified risk of bias criteria

**Biases**	**Domains of bias**	Y/N/UC	Risk of bias
Selection	Was the study population clearly specified and defined?		
	Did sampling methods increase the likelihood of a balanced and objective sample?		
	Did authors use explicit inclusion and exclusion criteria?		
	Did authors select samples that well represent the population?		
Measurement	Was the measurement tool shown to be reliable and valid?		
	Was the measurement tool piloted?		
	Was the scoring scheme adequately described?		
	Could participants have been influenced to respond in a certain way given the measurement techniques?		
Non-response	Was there the potential for non-response bias to influence results?		
	Did authors address possible non-response bias?		
Bias of data analysis	Were statistical analyses and reporting techniques appropriate to the data collected?		

<u>Selection bias:</u>

Low risk of bias – The study population was clearly specified and defined. Appropriate inclusion and exclusion criteria were applied uniformly. Probability sampling was employed to generate a balanced and objective random sample of the study population. Sampling methods adequately maximised generalisability and minimised sampling error.

Unclear risk of bias – The methods used to enrol participants nor the selection criteria for enrolment were clearly specified.

High risk of bias – The sampling methods applied were inadequate in ensuring a sample that was objective and balanced. There exists the potential that the sample was not representative of the study population. No consideration given for sampling error when performing statistical analysis.

Measurement bias:

Low risk of bias – The study uses a new or pre-existing measurement tool and adequately demonstrates that the tool has been previously assessed for validity and reliability. The tool was piloted by a subset of the study population prior to dissemination. The scoring scheme for the measurement tool was adequately described. Where applicable, methods adequately limit additional response bias.

Unclear risk of bias – Evidence for the tools validity and reliability was lacking; however, the study at least piloted the tool in a subset of the study population. Neither a description of the scoring scheme nor a discussion of methods used to limit response bias are clearly specified.

High risk of bias – The measurement tool was neither piloted nor demonstrates reliability and validity. The questionnaire design and measurement techniques used do not limit the potential for response bias. A description of the scoring scheme was not provided.

Non-response bias:

Low risk of bias – The response rate was adequately described and was considered robust. If the response rate was not considered robust, the study employed methods that addressed possible non-responder bias.

Unclear risk of bias – Information was insufficient to allow an assessment of whether non-response bias was influencing results. No response rate provided.

High risk of bias – The response rate was poor, and the study did not address this potential for bias. Alternatively, the study did perform analysis to address non-response bias but found noteworthy differences between responders and non-responders.

Bias of data analysis:

Low risk of bias – The study performs statistical analysis that is appropriate to the data collected (e.g. normal vs non-normal data). All analyses are purposeful, and the tests used are adequately described. The presentation of statistical results conforms to pre-existing convention. All results and discussions surrounding results are accurate and neutral in their presentation.

Unclear risk of bias – The study does not report the specifics of the statistical tests performed or does not provide enough information to make a sufficient decision as to the appropriateness of specific tests.

High risk of bias – The statistical tests used are not appropriate to the data collected. The presentation of results deviates from accepted standards. The reporting of results and discussions around these results do not maintain neutrality.
